# Occult Hepatitis C Virus Infection in Patients With Autoimmune Hepatitis

**DOI:** 10.5812/hepatmon.16089

**Published:** 2014-08-10

**Authors:** Mohammad Saeid Rezaee Zavareh, Seyed Moayed Alavian, Hamidreza Karimisari, Mostafa Shafiei, Seyed Yasser Saiedi Hosseini

**Affiliations:** 1 Students' Research Committee, Baqiyatallah University of Medical Sciences, Tehran, IR Iran; 2 Middle East Liver Diseases Center (MELD), Tehran, IR Iran

**Keywords:** Prevalence, Hepatitis C virus, Autoimmune Hepatitis, Infection, Diagnosis, Clinical Laboratory Technique

## Abstract

**Background:**

Occult hepatitis C virus infection (OCI) is recognized by finding hepatitis C virus (HCV) RNA in hepatocytes without detectable anti-HCV antibodies and viral RNA in plasma. Autoimmune hepatitis (AIH) is a chronic and generally progressive disease without exactly-identified etiology.

**Objectives:**

This study aimed to determine the prevalence of OCI among patients with AIH and to evaluate the tests used to rule out HCV infection in diagnosing AIH.

**Patients and Methods:**

Between July 2012 to February 2013, 35 Iranian patients with AIH who attended Tehran Hepatitis Center were investigated. For identifying OCI, detection of HCV RNA in both ultracentrifuged serum samples and peripheral blood mononuclear cells (PBMCs) was used. Data analysis was performed using SPSS.

**Results:**

Six males and 29 females with mean disease duration of 77.1 ± 39.5 month and mean age of 43.62 ± 12.67 years were investigated. All cases were negative for anti-HCV antibody and we could not find any HCV RNA in ultracentrifuged serum samples and PBMCs.

**Conclusions:**

With our laboratory diagnostic method, it seems that there are no cases of OCI in patients with AIH. However, we recommend further studies with more samples and more precise laboratory method.

## 1. Background

In 2004, Castillo et al. defined a new entity of chronic Hepatitis C virus (HCV) infection, ie, occult HCV infection (OCI), in which HCV RNA can be found in hepatocytes and in peripheral blood mononuclear cells (PBMCs) in nearly 70% of patients without detectable anti-HCV antibodies and viral RNA in plasma by usual tests (1, 2). Finding of HCV RNA in hepatocytes is the gold standard and the most precise method for diagnosing OCI; however, the liver biopsy is aggressive. Hence, we can use an alternative method that is recommended for diagnosis of OCI, which is finding of HCV RNA in PBMCs and in ultracentrifuged serum when liver biopsy is not available (3). OCI is reported in patient with cryptogenic liver disease, patients on hemodialysis, and family members of patients with OCI; however, it has been reported in healthy people without any liver disease too (1, 4).

OCI can result in minimal changes in liver tissue and although there are some reports of liver cirrhosis and hepatocellular carcinoma due to OCI, it is less severe than HCV infection is. It seems that all HCV genotypes (HCV-1 through HCV-6) can make OCI and it can occurs worldwide (5). Sometimes, differentiation between HCV infection and autoimmune hepatitis (AIH) is difficult (6). AIH is a chronic and generally progressive disease without any exactly-identified etiology, which is distributed worldwide and is more common in females than in males. It is diagnosed by histopathologic changes (interface hepatitis), clinical features, and elevated transaminase, immunoglobulins (Ig), and circulating autoantibodies (7, 8). Based on autoantibodies, AIH is categorized in two major form: type 1 is characterized by the presence of circulating antinuclear antibodies (ANA) and smooth muscle antibodies (SMA); and type 2 is determined by the presence of anti-liver-kidney microsomal 1 (LKM-1) and anti-liver cytosol 1 (ALC-1) antibodies (6).

Some viruses, particularly HCV, can induce autoimmune diseases (6) and HCV in some patients with chronic HCV infection can induce autoantibodies (ANA, SMA and anti-LKM-1) and consequently, autoimmune hepatitis (6, 9, 10). SMA, ANA, and with lower prevalence, anti-LKM-1 have been reported in a different populations with chronic HCV infection in different studies (10, 11). Moreover, AIH has been reported in some patients with HIV/HCV coinfection (12). 

There are similarities between cytochrome P450 (CYP) 2D6 (target antigen of LKM-1 antibody) sequence and HCV core; therefore, HCV core might have a role in molecular mimicry theory and may be a cause of autoimmune reaction and developing AIH type 2 (13). On the other hand, both HCV antibodies and HCV RNA can be found in a meaningful proportion of patients with AIH suggesting that HCV might have some role in autoimmune response and developing autoimmune hepatitis (13, 14).

Finally, another important aspect of the association between AIH and OCI is the existence of opposite therapeutic strategy for each one (6, 15). Although interferon, as an antiviral medication, is used for treatment of patients with HCV infection, it can have some effects on autoreactivity of T cells and therefore, after continued exposure to interferon, it can lead to induction of autoimmune reactions and developing or worsening of autoimmune hepatitis (16). In contrast, used corticosteroid for treatment of AIH can result in developing HCV infection (17).

## 2. Objectives

This study aimed to determine the prevalence of OCI among Iranian patients with AIH and with considering OCI, it aimed to evaluate the tests used to rule out HCV infection in diagnosing AIH.

## 3. Patients and Methods

### 3.1. Patients

Between July 2012 to February 2013, 35 Iranian patients with AIH, who attended Tehran Hepatitis Center, were enrolled in this descriptive cross-sectional study. Based on the defined criteria by the International Autoimmune Hepatitis Group (IAIHG) (18), their disease had been confirmed in our center prior to this study and they were under treatment.

### 3.2. Data Collection

We collected necessary data from each patient’s medical file in our database. They included some demographic information, laboratory tests including aspartate aminotransferase (AST), alanine aminotransferase (ALT), alkaline phosphatase (ALKP), platelet (PLT), serum γ-globulin (GGB), serum albumin, total and direct bilirubin, prothrombin time (PT), ferritin, IgG, IgA, IgM, and level of antibodies including ANA, SMA, LKM-1 (a titer of ≥ 1:40 was considered as positive result) (19). We also extracted stage and grade of liver biopsies for some patients with available results in our database.

### 2.3. OCI Identification

We used detection of HCV RNA in both ultracentrifuged serum samples and PBMCs. We obtained an informed consent form each patient. Our laboratory method for HCV RNA extraction and detection and OCI identification (primers, kits, etc) was similar to the study by Bokharaei-Salim et al. (1).

### 2.4. Data Analysis

Finally, data were analyzed using SPSS version 17 (SPSS Inc., Chicago, IL, USA). We expressed continuous variables in mean ± standard deviation (SD) and categorical variables in frequency (percentage).

## 4. Results

In our study, 35 patients with AIH (6 males and 29 females), mean disease duration of 77.1 ± 39.5 months, and mean age of 43.62 ± 12.67 years were evaluated. Three patients (8.6%) were smoker, 8.6% had a history of cirrhosis, and one patient (2.8%) was alcohol abuser. Twenty patients (57.1%) had fatigue, 24 (68.6%) had jaundice, 21 (60%) had pruritus, and 14 (40%) had myalgia. According to the available data on autoantibodies, ten patients (28.6%) had positive results for ASMA and 17 (48.6%) for ANA. In all cases, only two patients (5.7%) had positive results for both ANA and ASMA. One patient (2.8%) had positive results for LKM-1. The mean grade of liver biopsy was 6.5 ± 4.5 and mean stage was 3.2 ± 2.6. Mean of laboratory variables are shown in Table 1. All cases had negative results for anti-HCV antibody and we could not detect any HCV RNA in both ultracentrifuged serum samples and PBMCs.

**Table 1.** Patients' Laboratory Data ^a^

Laboratory Test	Mean ± SD
**AST, µ/L**	281.31 ± 373.79
**ALT, µ/L**	297.09 ±390.88
**ALKP, µ/L**	423.54 ± 352.67
**Platelet, 1000/mm**	231 ± 80
**Serum γ-globulin, g/l**	70.3 ± 104.3
**Serum albumin, g/l**	39.6 ± 05.3
**Total bilirubin, µmol/l**	46.86 ± 53.52
**Direct bilirubin, µmol/l**	20 ± 28.89
**PT, s**	13.40 ± 3.02
**Ferriti**	76.98 ± 68.17
**IgG, g/l**	17.24 ± 15.13
**IgA, g/l**	2.39 ± 1.34
**IgM, g/l**	1.56 ± 0.98

^a^ Abbreviations: AST, aspartate aminotransferase; ALT, alanine aminotransferase; ALKP, alkaline phosphatase; PT, prothrombin time; and Ig, immunoglobulin.

## 5. Discussion

To the best of our knowledge, it was the first report about prevalence of OCI in AIH patients. Until now, some studies have been conducted on OCI in patients with cryptogenic liver disease, patients on hemodialysis, and family members of patients with OCI. On the other hand, it has been shown that some patients with AIH are infected with occult hepatitis B virus (20). In this study, according to our diagnostic method, there were no cases of OCI in patients with AIH. It should be emphasized that our method for diagnosing OCI was detection of HCV RNA in both ultracentrifuged serum samples and PBMCs. The gold standard for diagnosis of OCI is probing HCV RNA in hepatocytes; however, the data on liver biopsy were not available for all patients and therefore, we used this suggested alternative method (3). It should be noted that at the time of study, all of the patients were under treatment for AIH.

For diagnosis of AIH, first we must rule out some disease, especially viral hepatitis like HCV infection by checking the anti-HCV antibody (18). Today, OCI is a cause of cryptogenic liver disease (1). Nevertheless, instead of assessing anti-HCV antibody, OCI is diagnosed with the aforementioned methods. According to the result of our study, there were no cases of OCI in patients with AIH and therefore, it seems that there may be no need to evaluate the OCI and usage of its diagnostic methods in AIH. However, two important issues should be taken into account: the sample size of our study and our diagnostic method, namely, detection of HCV RNA in PBMCs and in ultracentrifuged serum. It was reported that combination of these two diagnostic methods can lead to diagnosing of about 85% of OCI cases (3). Then, for making a better decision on using OCI diagnostic method in routine diagnostic workup of AIH, more studies with more sample size and more accurate diagnostic approaches are needed.
